# Bill Relating to Officers of Health

**Published:** 1903-03-21

**Authors:** 


### BILL RELATING TO OFFICERS OF HEALTH.

A Bill has been introduced into Parliament by Sir Francis Sharp Powell, backed by Sir Walter Foster, Mr. Talbot, Dr. Farquharson, Mr. Henry Hobhouse, Mr. Cripps, Mr. Heywood Johnstone, Sir Michael Foster, and Sir John Batty Tuke, to amend the law relating to the qualification and tenure of office of medical officers of health and inspectors, and to make further provisions relating to superannuation allowances for such officers and inspectors. The main objects of the Bill are to ensure that none but those properly qualified shall be appointed as officers of health, and to give them the same amount of security in their tenure of office as prevails in Scotland, and as Poor-law medical officers possess in England and medical officers of health here in the metropolis. It is also proposed to enable them by definite contribution from their salaries paid to a central fund to provide themselves with superannuation allowances as a provision for old age and in case of disablement. Provision is already made for Poor law medical officers. In the metropolis the sanitary inspector is designated such by law, but outside London the law names him "inspector of nuisances." The London designation, it is proposed, shall be that universally used.

Section 189 of the Public Health Act, 1875, gives power to urban authorities to appoint medical officers of health, surveyors, and inspectors of nuisances, and they may pay to the officers and servants so appointed or employed such reasonable salaries, wages, or allowances as the urban authority may think proper; and any such officer shall be removable by the urban authority at their pleasure. Section 190 gives the like power to rural authorities.

Section 108 of the Public Health (London) Act, 1891, provides that the Local Government Board shall have the same powers as they have in the case of a district medical officer of a poor law union with regard to the qualification, appointment, duties, salary, and tenure of office of every medical officer of health and sanitary inspector. A medical officer of health shall be removable by the sanitary authority with the consent of the Local Government Board or by that Board, and not otherwise. Provided that the Local Government Board shall take into consideration every representation made by the sanitary authority for the removal of any medical officer, whether based on the general interests of the district, on the conduct of such officer, or on any other ground.

With respect to Poor-law medical officers, it is provided by a general order of May 25, 1857, that every medical officer of a workhouse duly qualified at the time of his appointment according to the regulations of the Local Government Board then in force shall hold his office until he shall die or resign, or be proved to be insane by evidence which the Local Government Board shall deem sufficient, or become legally disqualified to hold such office, or be removed by the Local Government Board.

From the above it will be seen that while Poor-law medical officers and medical officers of health in London cannot be removed without the sanction of the Local Government Board medical officers of health and inspectors of nuisances in urban and rural districts are liable to dismissal at the pleasure of the bodies appointing them. It is very essential for the proper discharge of the duties of these offices that these officers should not be removable at the pleasure of urban and rural district councils.

The Bill now before Parliament provides that no person shall after the commencement of this Act be appointed an officer of health unless he either (1) is the holder of a certificate of the Local Government Board or of some body for the time being approved by that board that he has by examination shown himself competent for the office to which he is to be appointed; or (2) has for three consecutive years before the commencement of this Act served as an officer of health.

Officers of health to hold office till death, resignation, or insanity, or removal by or with the consent of the Local Government Board. In the case of temporary disability the salary of the medical officer shall not cease to be payable.

### Superannuation.

Every officer of health appointed by a local authority, whether before or after the commencement of the Act, who shall become incapable of discharging the duties of his office with efficiency by reason of permanent infirmity of mind or body, or of old age, who shall have completed an aggregate service of 35 years, or who shall have attained at the full age of 60 years, shall be entitled on resigning or otherwise ceasing to hold his office to receive during life a superannuation allowance according to the scale laid down in this Act, viz.:—An officer of health who has served for 10 years but less than 11 years shall be entitled to an annual allowance equal to fifteen-sixtieths of the average amount of his salary during the five years ending on the quarter day which immediately precedes the day on which he ceases to hold office, with an addition of one-sixtieth of such average amount for every additional completed year of service until the completion of a period of service of 35 years, when a maximum allowance of forty-sixtieths shall be granted. All services by an officer of health under any local authority shall be aggregated and reckoned, whether the service has been continuous or not, and whether his whole time has been devoted to the service or not. A local authority liable under this Act for a superannuation allowance in computing the amount of the allowance may, in consideration of peculiar professional qualifications or of special circumstances, with the consent of the Local Government Board add a number of years not exceeding 10 to the number of years which the officer to receive the allowance has actually served in the aggregate.

Where an officer of health has attained the age of 65 years, and the local authority under whom he shall then hold office are of opinion that it would be expedient in the interests of the public service that he should cease to hold office, it shall be competent for that local authority to require him to retire upon payment to him of the superannuation allowance to which he may be entitled under the Act.

Every superannuation allowance shall be payable to or in trust for the officer of health entitled thereto, and shall not be assignable or chargeable with his debts or other liabilities.

Subject to the provisions of this Act, every officer of health shall contribute annually, for the purposes of this Act, a percentage amount of his salary, according to the scale laid down by this Act, the amount to be from time to time deducted from his salary by the local authority. The percentage amounts to be deducted annually shall be as follows:—In case of officers with less than five years' service at, or appointed after the commencement of this Act, 2 per cent. of the salary for each year; in the case of officers with more than five and less than 15 years' service at the commencement of this Act, $2\frac{1}{2}$ per cent. of the salary for each year; in the case of officers with more than 15 years' service at the commencement of this Act, 3 per cent. of the salary of each year.

Any officer of health not appointed or re-appointed after the commencement of this Act may give notice of his intention not to avail himself of the superannuation, and in that case no deduction shall be made.

